# Cystine: A Key Protective Factor Against Childhood Hypo-HDL Cholesterolemia and Dyslipidemia—A Matched Case–Control Study

**DOI:** 10.3390/nu18101488

**Published:** 2026-05-07

**Authors:** Lianlong Yu, Qing Yue, Qianrang Zhu, Yiya Liu, Meina Tian, Changqing Liu, Zhenchuang Tang

**Affiliations:** 1 Shandong Center for Disease Control and Prevention, Jinan 250014, China; lianlong00a@163.com; 2 National Center for Women and Children’s Health, NHC, Beijing 100081, China; yueqing@ncwchnhc.org.cn; 3 Jiangsu Provincial Center for Disease Control and Prevention, Nanjing 210028, China; zhuqianrang@hotmail.com; 4 Guizhou Center for Disease Control and Prevention, Guiyang 550004, China; liuyiya163@163.com; 5 Hebei Center for Disease Control and Prevention, Shijiazhuang 050021, China; tianmei78715@163.com (M.T.); lcq93@126.com (C.L.); 6 Institute of Food and Nutrition Development, Ministry of Agriculture and Rural Affairs, Beijing 100081, China

**Keywords:** children, dyslipidemia, cystine, Hypo-HDL cholesterolemia, propensity score matching, machine learning, XGBoost

## Abstract

Background: Dietary cystine may influence lipid metabolism, but epidemiological evidence in children is limited. This study aimed to investigate the association between dietary cystine intake and dyslipidemia and its subtypes in Chinese children. Methods: Data were derived from the China National Nutrition and Health Surveillance of Children and Lactating Mothers (CNNHSCLM). After propensity score matching (1:1, caliper = 0.2), 3676 children aged 6–17 years (1838 with dyslipidemia, 1838 controls) were included. The Quantile g-computation (qgcomp) model assessed the joint effect of 20 amino acids. Multivariate logistic regression, subgroup analysis, restricted cubic splines (RCS), and five machine learning models (including XGBoost with Shapley Additive Explanation (SHAP) analysis) were applied to evaluate the association between cystine intake and dyslipidemia. Results: The qgcomp model showed that cystine had a negative weighting contribution to reducing the risk of hypo-HDL cholesterolemia. Multivariate logistic regression revealed that cystine intake was significantly negatively correlated with hypo-HDL cholesterolemia (OR = 0.67, 95%CI: 0.53–0.86, *p* = 0.002) and total dyslipidemia (OR = 0.84, 95%CI: 0.74–0.96, *p* = 0.010), but not with other subtypes. Subgroup analyses indicated interactions with BMI and sex. RCS showed a non-linear dose–response relationship for hypo-HDL cholesterolemia and a linear negative relationship for total dyslipidemia. The XGBoost model achieved the best predictive performance (AUC = 0.902), and SHAP analysis identified cystine as the most important feature inversely associated with dyslipidemia. Decision curve analysis confirmed its clinical net benefit. Conclusions: Dietary cystine intake is negatively associated with the risk of hypo-HDL cholesterolemia and total dyslipidemia in children, and cystine is an important negative correlate of dyslipidemia. These findings provide new scientific evidence for dietary prevention of dyslipidemia in children.

## 1. Introduction

Dyslipidemia is a common metabolic disorder in childhood, including types such as hypercholesterolemia, hypertriglyceridemia, mixed hyperlipoproteinemia, and hypo-HDL cholesterolemia [1]. In recent years, the prevalence of dyslipidemia in children has been on the rise [2], which is not only closely related to childhood obesity and insulin resistance, but also increases the risk of cardiovascular disease in adulthood [3,4,5]. Therefore, early identification and intervention of modifiable risk factors for dyslipidemia in children is of great public health significance.

Dietary nutrition is one of the key factors affecting lipid metabolism [6]. Previous studies have focused on the effects of macronutrients such as total fat, saturated fatty acids, and carbohydrates on blood lipids [7,8], while studies on amino acids, especially specific amino acids, and their association with dyslipidemia are relatively limited. Cystine, as the precursor of cysteine, supports the synthesis of glutathione and thereby reduces oxidative stress, which can damage the reverse transport of cholesterol and the function of HDL. Cystine is a sulfur-containing amino acid that participates in glutathione synthesis, redox balance, and lipid metabolism regulation [9,10,11].

Cystine, as the precursor of cysteine, supports the synthesis of glutathione and thereby reduces oxidative stress, which can damage the reverse transport of cholesterol and the function of HDL [9,10]. These pieces of evidence suggest that cystine plays a role in protecting the vascular endothelium of developing children. Basic research has shown that cystine can exert a metabolic negative correlation by modulating homocysteine metabolism, antioxidant defense, and liver lipid synthesis pathways [12,13]. However, epidemiological evidence in children is still insufficient, and existing studies are mostly limited to single amino acid analysis, lacking assessment of the combined effects of amino acid mixtures, and there are few studies that use machine learning methods to explore the importance of cystine in the prediction of dyslipidemia.

This study utilizes large-sample data from the China National Nutrition and Health Surveillance of Children and Lactating Mothers (CNNHSCLM) project, controls for confounding bias through propensity score matching [14], and uses the Quantile g-computation model to assess the combined effects of amino acid mixtures [15]. Furthermore, it employs multivariate logistic regression, subgroup analysis, restricted cubic splines, and various machine learning algorithms to systematically explore the association between dietary cystine intake and dyslipidemia and its subtypes in children. The aim is to provide a scientific basis for dietary prevention of dyslipidemia in children and to provide a reference for the formulation of subsequent nutritional intervention strategies.

## 2. Materials and Methods

### 2.1. Study Design and Population

The data for this study came from the China National Nutrition and Health Surveillance of Children and Lactating Mothers (CNNHSCLM) [16]. This survey used a multi-stage stratified cluster random sampling method, covering 275 monitoring sites in 31 provinces (autonomous regions and municipalities) across the country, and was conducted from 2016 to 2019. The study was approved by the Ethics Committee of the Institute of Nutrition and Health, Chinese Center for Disease Control and Prevention (approval number: 201614), and all participating children and their guardians signed written informed consent forms. This study was a cross-sectional study design, initially including 12,815 children aged 6 to 17 years from four provinces (Shandong, Jiangsu, Hebei, and Guizhou). The exclusion criteria were as follows: (1) 52 cases with missing BMI data were excluded; (2) 6 cases with missing gender data were excluded; (3) 15 cases with missing passive smoking exposure information were excluded; (4) 15 cases with missing drinking status information were excluded; (5) 51 cases with missing energy intake data were excluded; (6) 6 cases with missing cystine intake data were excluded; (7) 6 cases with missing blood lipid index data were excluded; (8) 27 cases with missing age information were excluded. After the above exclusions, in order to control confounding bias, the 1:1 Propensity Score Matching (PSM) method was adopted [14]. Dyslipidemia was used as the grouping variable, and age, gender, body mass index (BMI), outdoor activity time, passive smoking, alcohol consumption, and total energy intake were used as covariates. The caliper value was set to 0.2, and the nearest neighbor matching was performed. After matching, a total of 3676 participants were included in the study, including 1838 participants in the dyslipidemia group (case group) and 1838 participants in the non-dyslipidemia group (control group). The baseline characteristics of the two groups were balanced and comparable (standardized mean difference SMD < 0.1). PSM was employed to minimize confounding bias inherent in this observational cross-sectional study. In the initial sample, significant differences in several covariates (age, BMI, and alcohol consumption) were observed between the dyslipidemia and non-dyslipidemia groups. PSM with a caliper of 0.2 was therefore applied to create a balanced, matched case–control dataset (all standardized mean differences < 0.1), thereby enhancing group comparability and reducing potential confounding when estimating the association between dietary cystine intake and dyslipidemia.

### 2.2. Variable Definition and Measurement

Dietary intake data were collected using a food frequency questionnaire (FFQ) with verified reliability and validity [17]. The subjects were interviewed face-to-face by uniformly trained investigators to inquire about the frequency and amount of various foods consumed in the past month. At the same time, the weight of cooking oil and condiments from home or school canteens for three days was used to assist in the estimation. The daily intakes of amino acids (including 20 amino acids such as cystine) (g/day) and total energy, protein, fat and carbohydrates were calculated with reference to the Chinese Food Composition Table (Standard Edition 6) [18]. The definition of dyslipidemia is based on the “Expert Consensus on the Prevention and Treatment of Dyslipidemia in Chinese Children and Adolescents” [19]: Hypercholesterolemia (total cholesterol ≥ 5.18 mmol/L), Hypertriglyceridemia (triglyceride ≥ 1.70 mmol/L), mixed hyperlipoproteinemia (total cholesterol ≥ 5.18 mmol/L and triglyceride ≥ 1.70 mmol/L) and hypo-HDL cholesterolemia (HDL-C ≤ 1.04 mmol/L). Meeting any of the above criteria is considered dyslipidemia. Fasting venous blood samples were collected by professional nurses, and the four lipid items were detected by enzymatic method on a fully automated biochemical analyzer. Covariates included: age, gender, BMI, outdoor activity time (hours/day), passive smoking (yes/no), alcohol consumption (whether or not alcohol was consumed in the past 30 days), total energy intake (kcal/d), etc., all of which were obtained through standardized questionnaires or physical measurements. Total cholesterol (TC), triglycerides (TG), low-density lipoprotein cholesterol (LDL-C), and high-density lipoprotein cholesterol (HDL-C) were measured by a Hitachi Autoanalyzer 7600 (Hitachi, Tokyo, Japan). Anthropometric measurements were performed using standardized equipment: an electronic weight scale (G&G TC-200K, G&G Measurement Co., Ltd., Beijing, China) and an electronic blood pressure monitor (OMRON HBP1300, OMRON Healthcare Co., Ltd., Dalian, China), with measurement accuracies of 0.1 cm, 0.1 kg, and 1 mmHg, respectively. All lipid measurements were performed in a centralized laboratory of the Chinese Center for Disease Control and Prevention following strict standard operating procedures and internal quality control protocols, ensuring the accuracy and reliability of the results.

### 2.3. Statistical Analysis Methods

To reduce selection bias in observational studies, we used the PSM method [14]. Dyslipidemia was used as the outcome variable, and age, sex, BMI, outdoor activity time, passive smoking, alcohol consumption, and total energy intake were used as covariates. The propensity score of each individual was calculated using a logistic regression model. The 1:1 nearest neighbor matching method was used, and the caliper value was set to 0.2 to achieve balanced comparability between the case group and the control group. The standardized mean difference (SMD) of each covariate was calculated before and after matching, and the balance was judged to be achieved when SMD < 0.1 [20].

To assess the combined effects of a mixture of 20 amino acids with dyslipidemia and its subtypes, we employed a Quantile g-computation (qgcomp) model. This model converts the intake of each amino acid to an integer range of 0–3 based on quartiles, estimates the overall effect when all amino acids simultaneously increase by one quartile interval using a generalized linear model, and calculates the positive and negative weights of each amino acid to identify the component in the mixture that contributes most to the combined effect. The model was adjusted for age, sex, BMI, total energy intake, passive smoking, alcohol consumption, outdoor activity time.

Based on the significant combined effect of amino acid mixtures found in the qgcomp model (with cystine contributing negatively), multivariate logistic regression was used to analyze the association between each amino acid and dyslipidemia subtypes, with a focus on the results for cystine. Three adjusted models were constructed: Model 1 was the unadjusted crude model; Model 2 adjusted for age, sex, and BMI; Model 3 further adjusted for total energy intake, passive smoking, alcohol consumption, outdoor activity time. Results are expressed as odds ratios (OR) and their 95% confidence intervals (CI).

To investigate the association between cystine and low HDL cholesterol and dyslipidemia, subgroup analyses were conducted based on sex (male/female), age group (6–11 years/12–17 years), BMI (normal/overweight/obese), and protein intake level (low/high, stratified by median). Likelihood ratio tests were used to assess the interaction between stratification factors and cystine. Simultaneously, a restricted cubic spline (RCS) model was used to examine the nonlinear relationship between cystine intake and dyslipidemia risk, with three nodes (located at the 10th, 50th, and 90th percentiles), and model adjustments were performed similarly to Model 3.

To predict the risk of dyslipidemia and identify important predictive features, we constructed five machine learning models: Logistic Regression (LG), eXtreme Gradient Boosting (XGBoost), Light Gradient Boosting Machine (LightGBM), Naive Bayes (NB), and Neural Network (NN). In the PSM-matched samples, the performance of each model was evaluated using ten-fold cross-validation, with the area under the receiver operating characteristic curve (AUC-ROC) as the main evaluation index [21]. The best-performing XGBoost model was selected, and the contribution of each feature to the model output was calculated using the Shapley Additive Explanation (SHAP) algorithm. A beeswarm plot, a feature importance ranking plot, and a waterfall plot of individual predictions were generated. In addition, Decision Curve Analysis (DCA) was plotted to evaluate the clinical net benefit of the XGBoost model at different threshold probabilities, with standardized net benefit as the ordinate [22].

All statistical analyses were performed using R software (version 4.3.1), including the following R packages: MatchIt (PSM), qgcomp (Quantile g-computation), xgboost, lightgbm, e1071 (Naive Bayes), nnet (neural network), pROC (ROC curve), shapviz (SHAP analysis), and rms (RCS analysis). The Mann–Whitney U test was used for comparisons between groups of continuous variables, and the χ^2^ test was used for categorical variables. A two-tailed significance level of α = 0.05 was used, and *p* < 0.05 was considered statistically significant.

## 3. Results

### 3.1. Study Population and Propensity Score Matching Results

**Figure 1:**
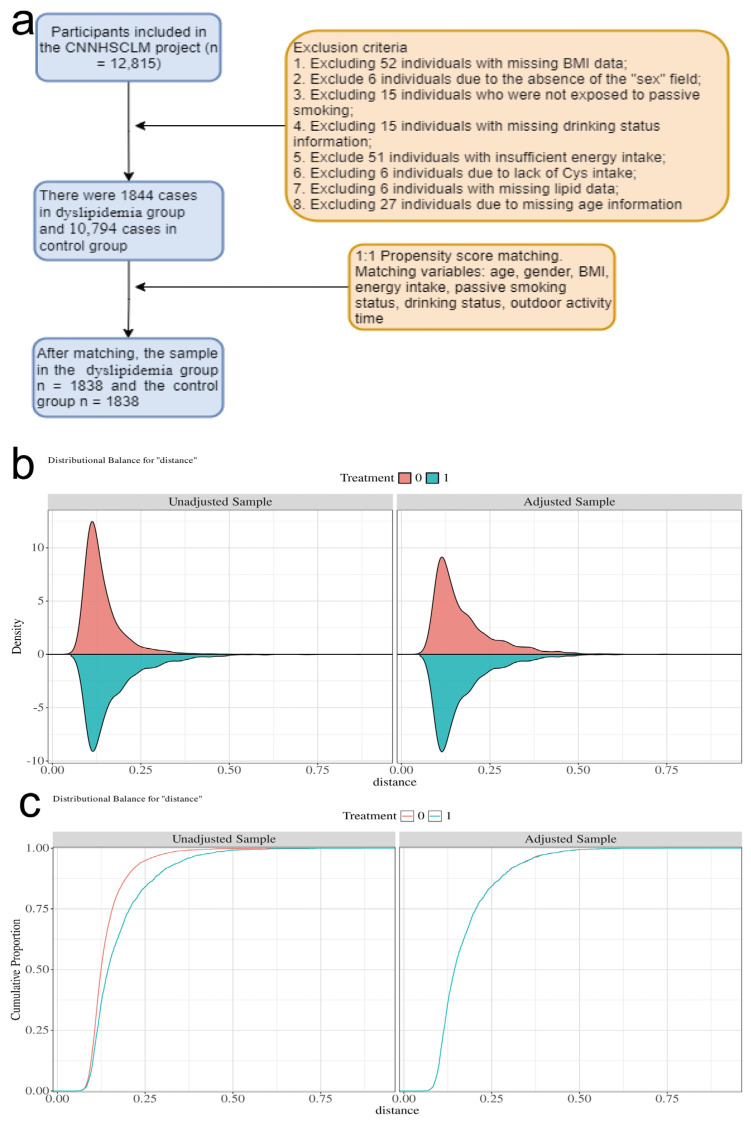
Study flow diagram and assessment of propensity score balance before and after matching. (**a**) Flowchart of participant selection from the CNNHSCLM project. (**b**) Histogram of propensity score distribution for the two groups in the adjusted sample (after matching), showing improved overlap. (**c**) Empirical cumulative distribution function (ECDF) of propensity scores for the unadjusted and adjusted samples, illustrating the balance achieved after matching.

This study initially included 12,815 children aged 6–17 years from the CNNHSCLM. Exclusion criteria were as follows: 52 cases with missing BMI data, 6 cases with missing gender data, 15 cases with missing passive smoking information, 15 cases with missing alcohol consumption status, 51 cases with abnormal energy intake, 6 cases with missing cystine intake, 6 cases with missing blood lipid data, and 27 cases with missing age information. A total of 12,638 valid samples were ultimately included. To control for confounding bias, dyslipidemia was used as the grouping variable, and a 1:1 propensity score matching (PSM) was employed with a caliper value of 0.2. 3676 individuals were successfully matched, with 1838 in the dyslipidemia group and 1838 in the non-dyslipidemia group (Figure 1a). Before matching, there were significant differences between the two groups in age, BMI, and alcohol consumption (*p* < 0.05); after matching, there were no statistically significant differences between the groups for any covariates (*p* > 0.05), and the absolute values of the SMD were all less than 0.1 (Table 1). The histogram of propensity score distribution (Figure 1b) shows that the two groups of scores overlap well after matching, and the empirical cumulative distribution function (ECDF) curve (Figure 1c) further confirms that the propensity score distributions of the two groups tend to be consistent after matching.

### 3.2. Baseline Characteristics of the Matched Population

A total of 3676 participants were included after matching, with a median age of 11.00 (9.00, 14.00) years and males accounting for 51.77% (1903/3676). There were no statistically significant differences between the dyslipidemia group and the control group in terms of age, sex, energy intake, BMI, protein intake, fat intake, carbohydrate intake, passive smoking, alcohol consumption, and outdoor activity time (*p* > 0.05), indicating that the two groups were comparable (Table 2). Spearman correlation analysis showed that cystine intake was significantly correlated with HDL-C (β = 0.0356, *p* = 0.0309) but not with LDL-C (β = 0.0099, *p* = 0.5477), while most other amino acids were positively correlated with both lipid parameters (*p* < 0.05).

### 3.3. Quantile g-Computation Analysis of Amino Acid Mixtures and Dyslipidemia

**Figure 2:**
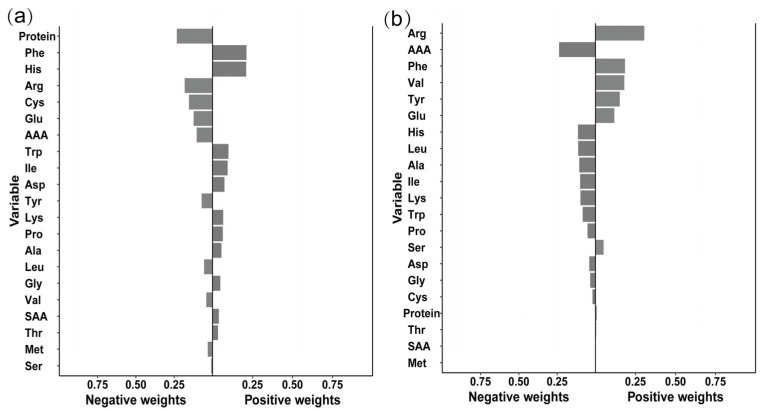
Quantile g-computation model for the association between amino acid mixture and lipid abnormalities. (**a**) Positive joint effect on hypercholesterolemia. A one-quantile increase in the amino acid mixture was associated with a 15.7% higher risk (OR = 1.157, 95% CI: 1.031–1.297, *p* = 0.013). The bar chart shows the positive and negative contribution weights for each amino acid in this model. (**b**) Negative joint effect on Hypo-HDL cholesterolemia. A one-quantile increase in the mixture was linked to a decreased risk (OR = 0.895, 95% CI: 0.809–0.989, *p* = 0.030). The corresponding weights for each amino acid are presented.

The combined effects of a mixture of 20 amino acids and dyslipidemia subtypes were assessed using a Quantile g-computation model. The results (Figure 2) showed that for every additional quartile interval in the amino acid mixture, the risk of hypercholesterolemia increased by 15.7% (OR = 1.157, 95%CI: 1.031–1.297, *p* = 0.013), with cystine showing a negative weighting (Figure 2a). For hypo-HDL cholesterol, for every additional quartile interval in the amino acid mixture, the risk decreased by 10.5% (OR = 0.895, 95%CI: 0.809–0.989, *p* = 0.030), with cystine also showing a negative weighting (Figure 2b). These findings suggest that cystine is the major contributor to reducing the risk of hypoHDL cholesterol in the amino acid mixture. The abbreviations are as follows: Ala—alanine; Arg—arginine; Asp—aspartic acid; Cys—cystine; Glu—glutamic acid; Gly—glycine; His—histidine; Ile—isoleucine; Leu—leucine; Lys—lysine; Met—methionine; Phe—phenylalanine; Pro—proline; Ser—serine; Thr—threonine; Trp—tryptophan; Tyr—tyrosine; Val—valine; SAA—sulfur-containing amino acids (methionine + cystine); AAA—aromatic amino acids (phenylalanine + tyrosine + tryptophan).

### 3.4. Association Between Cystine and Dyslipidemia Subtypes

Based on the negative contribution of cystine indicated by the qgcomp model, multivariate logistic regression was used to analyze the relationship between each amino acid and dyslipidemia. Only cystine showed a consistently stable statistical significance (Table 3). After fully adjusting the model (Model 3: adjusting for age, sex, BMI, total energy intake, passive smoking, alcohol consumption, and outdoor activity time), cystine intake was significantly negatively correlated with the risk of low HDL cholesterol (OR = 0.67, 95%CI: 0.53~0.86, *p* = 0.002) and also negatively correlated with the risk of total dyslipidemia (OR = 0.84, 95%CI: 0.74~0.96, *p* = 0.010). There was no statistically significant association between cystine and hypercholesterolemia, hypertriglyceridemia, or mixed hyperlipidemia (*p* > 0.05).

### 3.5. Subgroup Analysis, Interactions and Dose–Response Relationships

**Figure 3:**
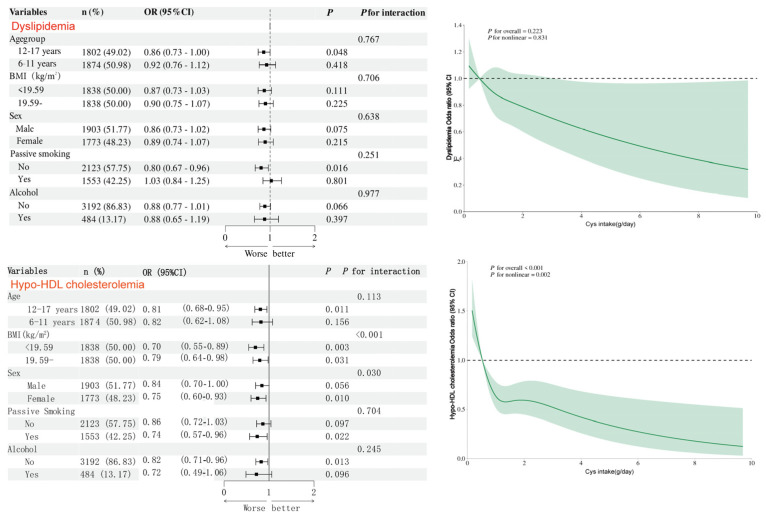
Subgroup analyses, interaction tests, and restricted cubic spline (RCS) curves for the association between cysteine (Cys) levels and dyslipidemia and hypo-HDL cholesterolemia. The solid green line is the multivariate adjusted odds ratio, and the shadow shows the 95% confidence interval from restricted cubic spline regression.

To investigate the association between cystine and low HDL cholesterol and total dyslipidemia, subgroup analyses were further conducted based on gender, age group, BMI, and protein intake level (Figure 3). Results showed that cystine exhibited a protective trend in all subgroups, and BMI and sex had an interaction effect on the relationship between cystine and hypo-HDL cholesterolemia (all interaction *p* values < 0.05). Restricted cubic spline (RCS) analysis revealed a linear negative correlation between cystine intake and the risk of dyslipidemia (non-linear *p* > 0.05), with a non-linear dose–response relationship with hypo-HDL cholesterolemia (non-linear *p* < 0.05) (Figure 3).

### 3.6. Predictive Performance of Machine Learning Models and Feature Importance

**Figure 4:**
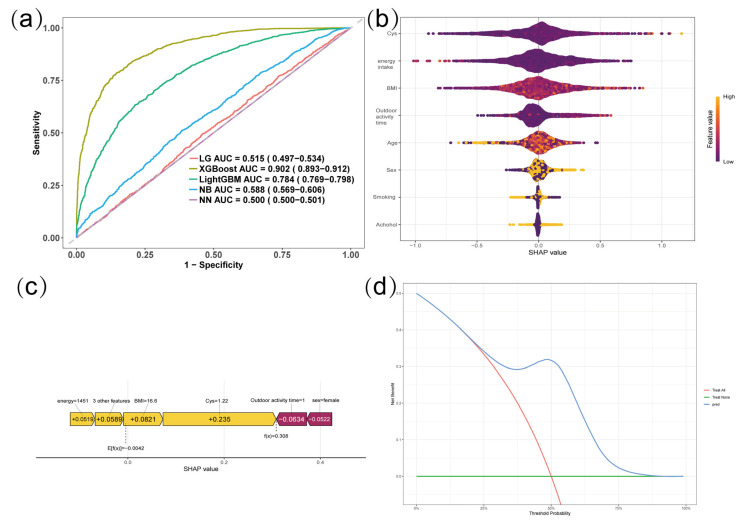
Performance comparison and model interpretability of machine learning algorithms for predicting dyslipidemia. (**a**) Receiver operating characteristic (ROC) curves of five models: LG (Logistic Regression), XGBoost, LightGBM, NB (Naïve Bayes), and NN (Neural Network). (**b**) SHAP summary (beeswarm) plot for the XGBoost model. Each point represents a SHAP value for a feature in an individual sample; the color indicates the feature value from low to high. The x axis shows the SHAP value (impact on model output), and the y axis lists the features in order of importance. (**c**) SHAP waterfall plot for a representative individual prediction (XGBoost model). The plot shows how each feature contributes to pushing the model’s predicted probability from the baseline value (expected value) to the final output. (**d**) Decision curve analysis (DCA) for the XGBoost model. The x axis shows the threshold probability, and the y axis shows the normalized net benefit (normalized density). The curve demonstrates the clinical utility of the XGBoost model across a range of threshold probabilities.

Five machine learning models (LG, XGBoost, LightGBM, NB, and NN) were constructed to predict the risk of dyslipidemia. The ROC curves of 10-fold cross-validation (Figure 4a) showed that the XGBoost model performed best, with an AUC of 0.902 (95% CI: 0.893–0.912), outperforming LightGBM (AUC = 0.784), NN (AUC = 0.500), LG (AUC = 0.515), and NB (AUC = 0.588). The Brier score of the XGBoost model was 0.048, indicating excellent calibration. The SHAP algorithm was used to interpret the XGBoost model. The SHAP beeswarm summary plot (Figure 4b) showed that among all included features, cystine had the largest absolute SHAP value, suggesting that cystine is the most important feature inversely associated with dyslipidemia. Figure 4c shows the SHAP waterfall plot for an individual’s prediction, indicating that cystine was the main feature driving the predicted probability below the baseline value. Decision curve analysis (DCA, Figure 4d) shows that the XGBoost model provides positive standardized net benefit over a wide threshold probability range (0.1–0.8), significantly outperforming the “full intervention” and “no intervention” strategies, demonstrating good clinical applicability.

## 4. Discussion

Based on large-sample data from the CNNHSCLM, this study systematically explored the association between dietary cystine intake and dyslipidemia and its subtypes in children using propensity score matching, quantile g-computation, multivariate logistic regression, subgroup analysis, restricted cubic splines, and various machine learning algorithms. The main findings are as follows: (1) Combined effect analysis of amino acid mixtures showed that cystine had a negative weighting contribution to reducing the risk of low HDL cholesterol; (2) Multivariate logistic regression confirmed that cystine intake was significantly negatively correlated with the risk of low HDL cholesterol and total dyslipidemia, but not statistically associated with high cholesterol, high triglycerides and mixed hyperlipidemia; (3) Subgroup analysis found that BMI and gender had an interactive effect on the relationship between cystine and low HDL cholesterol; (4) Dose–response relationship showed that cystine was non-linearly associated with low HDL cholesterol and linearly negatively correlated with total dyslipidemia; (5) XGBoost performed best in the machine learning model (AUC = 0.902), and SHAP analysis suggested that cystine was the most important negative correlate for predicting dyslipidemia.

There are very few epidemiological studies on the association between dietary cystine and blood lipids in children. Cystine is a sulfur-containing amino acid and a key precursor for glutathione synthesis and redox regulation [9,10,11]. Unlike previous studies focusing on branched-chain amino acids or total protein intake [23], the present study specifically examined cystine. Because dietary cystine and total protein are highly correlated (multicollinearity), conventional multivariate regression cannot reliably separate their individual effects. We therefore employed the Quantile g-computation (qgcomp) model, which is specifically designed to assess the joint effect of correlated mixtures and to identify the components that contribute most to the outcome [15]. In both the qgcomp analysis and the logistic regression analysis of 20 amino acids, cystine consistently showed a negative weighting contribution to reducing the risk of hypo-HDL cholesterolemia. In contrast, other amino acids showed either positive or negative weights in the qgcomp model, but their associations were not significant in the logistic regression analysis. These findings indicate that, within the amino acid mixture derived from dietary protein, cystine has a unique and robust inverse association that is not merely a proxy for total protein intake. Thus, the combination of qgcomp and logistic regression allowed us to disentangle the specific role of cystine from the overall protein matrix. The fact that cystine, but not several other amino acids with moderate mixture weights, remained significant in fully adjusted logistic regression indicates that its inverse association is more robust and independent, whereas other amino acids may act primarily through joint effects or are more susceptible to confounding. In adult studies, a cross-sectional and longitudinal metabolomics study from the Framingham Heart Study (discovery sample, *n* = 650; age 36–69 years) and the BioImage Study (replication sample, *n* = 670; age 61–76 years), with 554 participants followed for 5–7 years, used gas chromatography-mass spectrometry to measure plasma metabolites. Single-metabolite and multi-metabolite association tests as well as meta-analysis were applied. That study found that higher plasma levels of branched-chain amino acids and related metabolites (e.g., glutamic acid) were significantly associated with an increased risk of dyslipidemia [23]. There are also experimental animal studies that have examined the effects of *N*-acetylcysteine (NAC) supplementation on blood lipid profiles, but the results have been inconsistent. For example, a randomized controlled animal study [24] used male C57bl/6 mice fed a high-cholesterol diet for 8 weeks. NAC was administered orally at 230 mg/kg. Total cholesterol, LDL-cholesterol, HDL-cholesterol and triglycerides were measured, and the study found that NAC significantly reduced total and LDL-cholesterol levels compared to the high-cholesterol control group. In another animal study [25], 40 male Wistar rats were divided into four groups (*n* = 10 per group) and treated for 30 days with either standard chow, 2 mg/L NAC in drinking water, 30% sucrose, or sucrose plus NAC. Serum lipids, glucose, oxidative stress markers, and hepatic enzyme activities were assessed using analysis of variance (ANOVA) and appropriate post hoc tests. That study reported that NAC prevented sucrose-induced hyperglycaemia, dyslipidemia (elevated triglycerides, VLDL, and cholesterol/HDL ratio) and oxidative stress, and enhanced lipid degradation in hepatic tissue. The inconsistency between studies may be related to differences in baseline characteristics of the study population (e.g., mouse vs. rat, diet-induced hypercholesterolemia vs. sucrose-induced model), intervention dose and duration [24,25]. Compared with previous studies that only analyzed single amino acids, this study innovatively used the Quantile g-computation model to assess the combined effect of amino acid mixtures and found that cystine is one of the main contributors to reducing hypercholesterolemia and hypo-HDL cholesterolemia in the mixture. This methodological advantage makes the results closer to the synergistic effect of multiple amino acids in the real dietary environment. In addition, this study is the first to apply multiple machine learning algorithms to verify the importance of cystine in children. The XGBoost model showed good predictive efficacy, and SHAP analysis further confirmed the key role of cystine. This provides a new idea for nutritional epidemiology research in terms of methodology.

This study found a significant interaction between BMI and sex on the relationship between cystine and hypo-HDL cholesterolemia. The BMI interaction suggests that the negative correlation of cystine may vary depending on body weight: the negative correlation of cystine is more pronounced in children with lower BMI; while in children with higher BMI, the metabolic transformation of [26], thus weakening its negative correlation, but still significant. The sex interaction may be related to differences in estrogen levels. Female children experience increased estrogen levels after puberty [27]. Estrogen itself has antioxidant and HDL metabolism-improving effects, which may have a synergistic or antagonistic effect with cystine, leading to differences in effects between sexes [28,29,30]. This finding suggests that future dietary interventions targeting cystine should consider individual BMI and sex factors to develop more precise nutritional strategies. In addition, RCS analysis showed a non-linear relationship between cystine and low HDL cholesterol, suggesting a possible threshold effect, where the negative correlation tends to plateau after a certain intake level. This provides a reference for determining the appropriate cystine intake range for children.

This study compared the performance of five machine learning models in predicting dyslipidemia. The XGBoost model achieved the best predictive performance (AUC = 0.902, 95%CI: 0.893–0.912), substantially outperforming traditional logistic regression (AUC = 0.515), LightGBM (AUC = 0.784), and Naive Bayes (AUC = 0.588). This indicates that XGBoost is better able to capture non-linear relationships and complex interactions among dietary, demographic, and lifestyle variables—such as those between cystine, BMI, sex, and age—which conventional regression models may miss. It should be noted that the high AUC (0.902) and low Brier score (0.048) were obtained from internal ten-fold cross-validation. While cross-validation reduces overfitting risk, external validation in independent cohorts is still required before clinical application. Therefore, the current machine learning findings should be interpreted as exploratory. The SHAP algorithm provided interpretable, subject-level explanations of the model’s predictions, which is particularly valuable in pediatric nutritional epidemiology where understanding individual risk profiles is clinically relevant. SHAP analysis confirmed that cystine was the most important protective feature, identifying it in a data-driven manner and extending our regression-based results. Decision curve analysis further confirmed that the XGBoost model could provide positive clinical net benefit over a wide range of threshold probabilities (0.1–0.8), indicating its practical application potential [31]. In the future, a simplified prediction tool can be constructed based on the feature importance ranking from this study for screening the risk of dyslipidemia in children. Overall, machine learning models, and XGBoost in particular, are well-suited to handle the high-dimensional, correlated nature of amino acid intake data in children and offer advantages for identifying key nutritional predictors in complex observational datasets. Nevertheless, LightGBM also showed acceptable performance (AUC = 0.784), and logistic regression retains value for its interpretability; the choice of model should depend on the specific analytic goal.

Beyond overall predictive performance, our XGBoost model offers important advantages for individual-level risk assessment. As shown in Figure 4c, the SHAP waterfall plot for a representative child clearly demonstrates how each feature, particularly cystine intake, contributes to the predicted probability of dyslipidemia. This ability to provide interpretable, personalised risk profiles is clinically valuable, as it allows paediatricians to identify children who would most benefit from dietary counselling or further testing. Thus, even before external validation in other populations, the model has practical utility in the Chinese paediatric population from which it was derived. Nevertheless, generalisability to other ethnic groups, age ranges, or geographic regions requires external validation. Differences in dietary patterns, genetic background, and lifestyle factors may affect model performance. Therefore, future studies should validate our XGBoost model in independent, diverse cohorts before widespread clinical implementation. Transfer learning or model recalibration may also be needed when applying the model to populations with substantially different characteristics. In the meantime, the current framework and the SHAP-based individual prediction approach can serve as a template for building similar tools in other settings.

Our XGBoost model has the potential to become a practical clinical tool for paediatricians. As shown in Figure 4c, it already enables interpretable, individual-level risk prediction. After external validation, a simplified version could be embedded into electronic health records or developed as a web-based calculator. Using readily available inputs (e.g., cystine intake, age, sex, BMI, outdoor activity time), paediatricians could screen children at high risk of dyslipidemia, facilitating early dietary counselling. Future studies are needed to confirm the causal effect of cystine supplementation and define optimal intake thresholds.

Cystine is a sulfur-containing amino acid that can be reduced to cysteine in vivo, which then participates in glutathione synthesis and homocysteine metabolism [32,33]. This study found that cystine is inversely associated with low HDL cholesterol and total dyslipidemia. Based on previous experimental studies, the following evidence-based mechanisms may explain the observed inverse association with hypo-HDL cholesterolemia. First, cystine is a precursor to glutathione synthesis. Sufficient glutathione can enhance the body’s antioxidant capacity, reduce the interference of oxidative stress on lipoprotein metabolism, and thus maintain the normal synthesis and function of HDL [34,35]. Second, cystine can reduce endothelial damage and lipid peroxidation caused by hyperhomocysteinemia by affecting the homocysteine metabolic pathway [36]. Third, as a hypothesis-generating statement, animal experiments have shown that sulfur-containing amino acids can regulate the expression of apolipoprotein AI (apoA-I) in the liver, and apoA-I is a major protein component of HDL. Its elevated level helps maintain HDL-C [37,38]. In addition, cystine may also indirectly improve lipid metabolism disorders by activating the nuclear factor E2-related factor 2 (Nrf2) pathway and upregulating the expression of antioxidant enzymes [39]. These mechanisms are plausible but were not directly tested in our study. However, in this study, cystine did not show a significant association with hypercholesterolemia or hypertriglyceridemia, suggesting that the effect of cystine may be specifically focused on the HDL metabolic pathway rather than comprehensively affecting all lipid components. Cystine was associated with hypo-HDL cholesterolemia but not with hypercholesterolemia or hypertriglyceridemia, suggesting a pathway-specific effect rather than global lipid modulation. This specificity may be explained by the particular sensitivity of HDL to oxidative stress. HDL particles carry antioxidant enzymes such as paraoxonase-1, and oxidative damage impairs HDL function and reverse cholesterol transport [34,35]. Cystine, as a precursor of glutathione, helps maintain intracellular redox balance, thereby protecting HDL from oxidative modification. In contrast, LDL and triglyceride metabolism are more strongly influenced by dietary fat and carbohydrate balance, which may explain the absence of association with cystine. This redox-mediated pathway supports a specific role of cystine in HDL metabolism.

In summary, dietary cystine intake was negatively correlated with the risk of hypo-HDL cholesterolemia and Dyslipidemia in children, and cystine was the most important feature for predicting dyslipidemia by machine learning models. This suggests that appropriately increasing the intake of cystine-rich foods (such as poultry, eggs, beans, whole grains, etc.) [18] may help improve HDL metabolism in children and reduce the risk of dyslipidemia. For clinicians, it is recommended that children with dyslipidemia pay attention to the intake of sulfur-containing amino acids on the basis of a balanced diet. For public health policymakers, dietary recommendations related to cystine can be included in the guidelines for promoting cardiovascular health in children. Considering the interaction between BMI and gender, overweight or obese children and male children may need more personalized nutritional intervention programs. Future research should further explore the action threshold of cystine and its synergistic effects with other nutrients to provide a scientific basis for precision nutrition.

Strengths of this study include the following. First, this study drew on large-scale, multicenter data from a national nutrition and health survey of children and lactating mothers, ensuring high representativeness of the pediatric population in China. Second, propensity score matching was employed to minimize confounding bias, thereby enhancing the comparability between groups. Third, the study innovatively integrated Quantile g-computation with multiple machine learning algorithms, allowing a two-dimensional evaluation of cystine’s role—both as part of a mixture’s joint effect and as an individual predictor. Fourth, comprehensive sensitivity analyses, including subgroup analyses, interaction tests, and dose–response assessments, were performed, yielding robust and consistent results.

This study also has certain limitations: First, the study design was a case–control study, which cannot infer a causal relationship between cystine intake and dyslipidemia. Second, dietary data were collected through a food frequency questionnaire (FFQ), which is subject to recall bias and potential misclassification of amino acid intake. Additionally, the FFQ did not collect detailed information on specific dietary patterns (e.g., Western vs. traditional) or the main food sources of cystine. Therefore, residual confounding by unmeasured dietary factors cannot be completely excluded. Third, although the PSM adjusted for major confounding factors, residual confounding factors may still exist (such as genetic factors, detailed measurement of physical activity, etc.); fourth, the study subjects were Chinese children, and extrapolation of the conclusions to other ethnic groups or age groups should be approached with caution. Future prospective cohort studies and randomized controlled trials are needed to further verify the causal effect of cystine on lipid metabolism in children and the appropriate intake level.

## 5. Conclusions

Dietary cystine intake was significantly negatively correlated with the risk of hypo-HDL cholesterolemia and dyslipidemia in children, and cystine was the main contributor to reducing hypo-HDL cholesterolemia in the amino acid mixture. BMI and sex interacted with these relationships, and the relationship between cystine and Hypo -HDL cholesterolemia showed a non-linear dose–response relationship. The XGBoost machine learning model confirmed that cystine is the most important negative correlate in predicting dyslipidemia, and the XGBoost model demonstrated good predictive efficacy and net clinical benefit. This study provides new scientific evidence for dietary prevention of dyslipidemia in children and suggests that cystine intake should be considered within a reasonable dietary framework.

## Figures and Tables

**Table 1:** Baseline graphs before and after propensity score matching.

Variable	Before PSM						After PSM					
	Total (*n* = 12,638)	0 (*n* = 10,794)	1 (*n* = 1844)	Statistic	*p*	SMD	Total (*n* = 3676)	0 (*n* = 1838)	1 (*n* = 1838)	Statistic	*p*	SMD
Outdoor activity time (hour/day), M (Q_1_, Q_3_)	1.33 (1.00, 2.00)	1.33 (1.00, 2.00)	1.33 (1.00, 2.00)	Z = −1.367	0.172	−0.024	1.33 (1.00, 2.00)	1.33 (1.00, 2.00)	1.33 (1.00, 2.00)	Z = −0.669	0.504	−0.040
Age, M (Q_1_, Q_3_)	11.00 (9.00, 14.00)	11.00 (8.00, 13.00)	11.00 (9.00, 14.00)	Z = −4.299	<0.001	0.106	11.00 (9.00, 14.00)	11.00 (9.00, 14.00)	11.00 (9.00, 14.00)	Z = −0.095	0.925	0.002
BMI(kg/m^2^), M (Q_1_, Q_3_)	18.09 (15.97, 20.94)	17.90 (15.89, 20.55)	19.56 (16.71, 23.28)	Z = −15.255	<0.001	0.383	19.58 (16.65, 23.23)	19.62 (16.61, 23.23)	19.54 (16.70, 23.23)	Z = −0.045	0.964	0.000
Energy2, M (Q_1_, Q_3_)	1821.07 (1297.87, 2600.37)	1821.00 (1298.86, 2603.63)	1823.31 (1292.01, 2585.22)	Z = −0.101	0.919	0.000	1826.33 (1294.08, 2598.70)	1827.48 (1296.08, 2609.24)	1821.54 (1291.82, 2586.21)	Z = −0.480	0.631	0.001
Sex, *n* (%)				χ^2^ = 2.507	0.113					χ^2^ = 0.001	0.974	
1	6323 (50.03)	5369 (49.74)	954 (51.74)			0.040	1903 (51.77)	952 (51.80)	951 (51.74)			−0.001
2	6315 (49.97)	5425 (50.26)	890 (48.26)			−0.040	1773 (48.23)	886 (48.20)	887 (48.26)			0.001
Passive smoking, *n* (%)				χ^2^ = 2.434	0.119					χ^2^ = 0.028	0.867	
0	7487 (59.24)	6425 (59.52)	1062 (57.59)			−0.039	2123 (57.75)	1064 (57.89)	1059 (57.62)			−0.006
1	5151 (40.76)	4369 (40.48)	782 (42.41)			0.039	1553 (42.25)	774 (42.11)	779 (42.38)			0.006
Alcohol, *n* (%)				χ^2^ = 12.969	<0.001					χ^2^ = 0.152	0.696	
0	11,295 (89.37)	9691 (89.78)	1604 (86.98)			−0.083	3192 (86.83)	1592 (86.62)	1600 (87.05)			0.013
1	1343 (10.63)	1103 (10.22)	240 (13.02)			0.083	484 (13.17)	246 (13.38)	238 (12.95)			−0.013

**Table 2:** Baseline characteristics and differences analysis.

Variables	Total (*n* = 3676)	Control Group (*n* = 1838)	Dyslipidemia Group (*n* = 1838)	Statistic	*p*
Age (years), M (Q_1_, Q_3_)	11.00 (9.00, 14.00)	11.00 (9.00, 14.00)	11.00 (9.00, 14.00)	Z = −0.09	0.925
Sex, n(%)				χ^2^ = 0.00	0.974
Male	1903 (51.77)	952 (51.80)	951 (51.74)		
Female	1773 (48.23)	886 (48.20)	887 (48.26)		
Energy intake (kcal/day), M (Q_1_, Q_3_)	1826.33 (1294.08, 2598.70)	1827.48 (1296.08, 2609.24)	1821.54 (1291.82, 2586.21)	Z = −0.48	0.631
BMI(kg/m^2^), M (Q_1_, Q_3_)	19.58 (16.65, 23.23)	19.62 (16.61, 23.23)	19.54 (16.70, 23.23)	Z = −0.04	0.964
Protein intake, M (Q_1_, Q_3_)	103.39 (70.53, 149.77)	103.85 (70.37, 150.56)	102.79 (71.11, 148.90)	Z = −0.07	0.946
Fat intake, M (Q_1_, Q_3_)	33.33 (19.59, 56.80)	33.80 (19.47, 58.29)	32.84 (19.71, 55.87)	Z = −0.45	0.653
Carbohydrate intake (g/day), M (Q_1_, Q_3_)	304.23 (218.20, 426.56)	306.68 (218.45, 429.52)	302.45 (218.01, 424.47)	Z = −0.50	0.620
Passive smoking, *n* (%)				χ^2^ = 0.03	0.867
No	2123 (57.75)	1064 (57.89)	1059 (57.62)		
Yes	1553 (42.25)	774 (42.11)	779 (42.38)		
Alcohol, *n* (%)				χ^2^ = 0.15	0.696
No	3192 (86.83)	1592 (86.62)	1600 (87.05)		
Yes	484 (13.17)	246 (13.38)	238 (12.95)		
Outdoor activity time (hour/day), M (Q_1_, Q_3_)	1.33 (1.00, 2.00)	1.33 (1.00, 2.00)	1.33 (1.00, 2.00)	Z = −0.67	0.504
Cys intake (g/day), M (Q_1_, Q_3_)	1.31 (0.92, 1.87)	1.33 (0.93, 1.90)	1.30 (0.91, 1.85)	Z = −0.88	0.381

Z: Mann–Whitney test, χ^2^: Chi-square test. M: Median, Q_1_: 1st Quartile, Q_3_: 3st Quartile.

**Table 3:** Cystine and risk of dyslipidemia subtypes.

	Model 1		Model 2		Model 3	
	*p*	OR (95%CI)	*p*	OR (95%CI)	*p*	OR (95%CI)
Hypercholesterolemia	0.304	0.97 (0.92–1.03)	0.062	1.12 (0.99–1.25)	0.554	1.06 (0.88–1.27)
Hypertriglyceridemia	0.49	0.96 (0.87–1.07)	0.239	0.93 (0.83–1.05)	0.158	0.83 (0.65–1.07)
mixed hyperlipoproteinaemia	0.22	0.80 (0.56–1.14)	0.241	0.78 (0.51–1.18)	0.109	0.49 (0.21–1.17)
Hypo-HDL cholesterolemia	0.49	0.96 (0.87–1.07)	0.002	0.86 (0.78–0.94)	0.002	0.67 (0.53–0.86)
Dyslipidemia	0.22	0.80 (0.56–1.14)	0.265	0.97 (0.91–1.03)	0.010	0.84 (0.74–0.96)

## Data Availability

Since this study data copyright belongs to the Chinese center for disease control, if has the need to share data and code please contact the first author and corresponding author, or visit https://www.chinacdc.cn/.
